# Human performance data collected in a virtual environment

**DOI:** 10.1016/j.dib.2017.09.029

**Published:** 2017-09-22

**Authors:** Mashrura Musharraf, Jennifer Smith, Faisal Khan, Brian Veitch, Scott MacKinnon

**Affiliations:** a Centre for Risk, Integrity and Safety Engineering (C-RISE), Faculty of Engineering & Applied Science, Memorial University of Newfoundland, St John's, Newfoundland and Labrador, Canada A1B 3X5; b Department of Mechanics and Maritime Sciences, Chalmers University, Gothenburg, Sweden

## Abstract

This data article describes the experimental data used in the research article “Incorporating individual differences in human reliability analysis: an extension to the virtual experimental technique” (Musharraf et al., 2017) [1]. The article provides human performance data for 36 individuals collected using a virtual environment. Each participant was assigned to one of two groups for training: 1) G1: high level training and 2) G2: low level training. Participants’ performance was tested in 4 different virtual scenarios with different levels of visibility and complexity. Several performance metrics of the participants were recorded during each scenario. The metrics include: time to muster, time spent running, interaction with fire doors and watertight doors, interaction with hazards, and reporting at different muster locations.

**Specifications Table**

**Table:** 

Subject area	*Engineering, Human factors*
More specific subject area	*Safety & Risk, Human Reliability Analysis*
Type of data	*Text files*
How data was acquired	Data were collected by conducting an experiment in a virtual environment. The virtual environment used is called the all-hands virtual emergency response trainer (AVERT) and was developed at the Memorial University. AVERT was designed to enhance offshore emergency response training. The virtual environment is modeled after an offshore oil installation platform with high levels of detail. It is capable of creating credible emergency scenarios by introducing hazards such as blackouts, fires and explosions.
	Human performance data of 36 individuals tested in simulated emergency scenarios in AVERT were collected.
Data format	*Filtered and processed*
Experimental factors	*The participants were naïve concerning any detail of the experimental design, they were not employed in the offshore oil and gas industry, and were not familiar with the AVERT simulator prior to the experiment. Their ages ranged from 19–39 years. Information regarding participants’ gaming and marine experience was collected prior to the experiment. This information guided the assignment of participants into different training groups. Participants were provided with basic offshore emergency preparedness training tutorials before performing in any simulated emergency scenarios.*
Experimental features	*Two performance shaping factors (PSFs) – visibility and complexity – were each tested at two different levels to create*$2^{2}=4$*virtual testing scenarios. Participants’ performance was tested in the scenarios and the following performance metrics were collected: time to muster, time spent running, interaction with fire doors and watertight doors, interaction with hazards, and reporting at different muster locations (i.e. mess hall/muster station, lifeboat starboard side, lifeboat port side).*
Data source location	*Memorial University of Newfoundland, St. John's, NL, Canada*
Data accessibility	*The data are with this article.*

**Value of the data**

• The data serve as a benchmark for human performance in emergency situations.
• The data allow objective assessment of human reliability rather than subjective assessments that rely on expert judgement.
• The data enable investigating the effects of different PSFs on human performance.
• The data provide the information that each human is different and the effect of PSFs on performance can vary from individual to individual.
• Analysis of the data can provide direction towards adaptive training.

## 1 Data

Human performance data for 36 individuals in 4 testing scenarios are associated with this article. The testing scenarios were created in AVERT. Two PSFs - visibility and complexity – were varied in the scenarios. Details of the 4 testing scenarios can be found in Table 3 in [1].

Performance metrics recorded during the scenarios include: time to muster, time spent running, interaction with fire doors and watertight doors, interaction with hazards, and reporting at different muster locations.

The 4 supplementary text files summarize the performance metrics of 36 individuals in the 4 simulated emergency scenarios.

## 2 Experimental design, materials and methods

The data presented in this article were originally collected during an experimental study presented in [2], [3]. Though a broad range of human performance data were collected during the study, this article only presents the data relevant to the article “Incorporating individual differences in human reliability analysis: an extension to the virtual experimental technique” [1].

A total of 36 participants took part in the study with a goal to learn how to perform a successful offshore emergency evacuation. The participants were naïve concerning any detail of the experimental design, they were not employed in the offshore oil and gas industry, and therefore they were not familiar with the offshore platform. Each participant was assigned to one of two groups for training: 1) G1: high level training and 2) G2: low level training. Participants in both groups received basic offshore emergency preparedness training. Participants in G1 received additional training tutorials and practice scenarios on alarms and hazards.

Once a participant was assigned to a group, his/her training level remained static (either low or high) for the rest of the study. The PSFs visibility and complexity, on the other hand, were set to different levels to investigate how these PSFs influence each participant. The schematic diagram of the experimental design can be found in [1].

## References

[1] Musharraf M., Smith J., Khan F., Veitch B., MacKinnon S. (2017). Incorporating individual differences in human reliability analysis: an extension to the virtual experimental technique. Saf. Sci..

[2] Smith J. (2015). The Effect of Virtual Environment Training on Partcipant Competence and Learning in Offshore Emergency Egress Scenarios.

[3] Musharraf M., Smith J., Khan F., Veitch B., MacKinnon S. (2016). Assessing offshore emergency evacuation behavior in a virtual environment using a Bayesian Network approach. Reliab. Eng. Syst. Saf..

